# Between Recognition and Belonging: Gifted Adults with a Migration Background Across the Lifespan

**DOI:** 10.3390/bs16101851

**Published:** 2026-10-09

**Authors:** Fatima Aitouny, Othilie Grisez, Zinat Maftun, Lianne Hoogeveen

**Affiliations:** Behavioural Science Institute (BSI), Radboud University, 6525 XZ Nijmegen, The Netherlands; othilie.grisez@ru.nl (O.G.); zinat.maftun@ru.nl (Z.M.); lianne.hoogeveen@ru.nl (L.H.)

**Keywords:** gifted adults, migration background, lifespan perspective, identity development, talent development, belonging, cultural context, under-recognition

## Abstract

This study examines how giftedness is experienced by adults with a migration background, addressing a gap in the literature concerning their experiences across the lifespan. Drawing on talent development perspectives and Bronfenbrenner’s ecological systems theory, giftedness is conceptualized as a context-dependent developmental process shaped by sociocultural environments. Data were collected through in-depth, semi-structured interviews and focus group discussions and analysed using an iterative, primarily inductive thematic analysis. The findings indicate that giftedness is not consistently recognized across contexts, but that its recognition varies in relation to educational practices, cultural expectations, and social positioning. Participants described recurring experiences of under-recognition and the need to validate their abilities, reflecting how dominant norms influence the identification of giftedness. In addition, the results highlight the central role of belonging and identity, with participants navigating multiple contexts and experiencing persistent misalignment. For some participants, additional identity-related dimensions, such as adoption and religion, added further layers to processes of meaning-making. The study contributes to the literature by highlighting how experiences of giftedness, recognition, belonging, and identity are embedded within sociocultural contexts and unfold across the lifespan. These findings underline the need for more context-sensitive and inclusive models of giftedness.

## 1. Introduction

Giftedness has traditionally been conceptualized as an individual attribute, often associated with high cognitive ability and exceptional performance. Although contemporary models increasingly emphasize developmental and multidimensional perspectives, such as talent development frameworks, questions remain about how sociocultural contexts are involved in the recognition and development of giftedness, particularly in migration-related contexts (Bicakci & Ziegler, 2026; Preckel et al., 2020; Subotnik et al., 2012, 2021). However, comparatively less is known about how these processes are experienced across the lifespan by gifted adults with a migration background (Brown et al., 2020; Rinn, 2024).

This limitation becomes particularly visible in the context of migration. Research has documented inequities in the identification and educational opportunities of high-ability students from minoritized and migration backgrounds (Ford, 2014; Ford et al., 2026; Gray & Gentry, 2024; Parekh et al., 2018; Ricciardi et al., 2020). Recent research in the Netherlands similarly found discrepancies between measured high ability and teacher and parent nominations, with identification varying according to factors including language and migration background (Hovinga & Cramer, 2026). These disparities reflect broader processes in which dominant cultural norms shape definitions of ability and merit (Parekh et al., 2018). These inequalities not only affect access to educational opportunities but may also influence broader developmental processes related to recognition, belonging, and motivation across the lifespan (Geurts et al., 2021; Rinn, 2024; Rinn & Bishop, 2015). Taken together, this literature suggests that the recognition of giftedness is not independent of the social and institutional contexts in which abilities are interpreted (Ford, 2014; Hovinga & Cramer, 2026; Parekh et al., 2018).

At the same time, migration-related experiences introduce additional developmental complexity. Individuals navigating multiple cultural contexts often face challenges related to belonging, identity formation, and social positioning (Geurts et al., 2021; Schachner et al., 2018). A sense of belonging has been identified as a fundamental psychological condition influencing well-being, identity development, and social functioning (Hagerty et al., 1992). Experiences of discrimination can further affect well-being and developmental trajectories (Alegria et al., 2019; Chen et al., 2020). For individuals with high ability, these dynamics may be particularly relevant to understanding social–emotional and developmental experiences across different contexts and stages of life (Rinn, 2024; Rinn & Bishop, 2015).

Bronfenbrenner’s ecological systems theory and later bioecological developments (Bronfenbrenner, 1979; Bronfenbrenner & Morris, 2006) provide a framework for understanding these dynamics by situating development within interacting environmental systems. Complementing this perspective, talent development frameworks emphasize how access to opportunities and support structures shapes the realization of potential over time (Gagné, 2004; Preckel et al., 2020; Subotnik et al., 2012). Together, these frameworks suggest that giftedness cannot be understood independently of the contexts in which it develops (Sternberg, 2024).

Despite these advances, research on giftedness in migration-related contexts has predominantly focused on children and adolescents, particularly in relation to identification, education, and access to support (Bicakci & Ziegler, 2026; Henfield et al., 2017; Mehdipour Maralani & Pfeiffer, 2025). Comparatively less is known about how gifted adults with a migration background experience recognition, belonging, and identity across the lifespan. A narrative review of the literature (El Hasnaoui-Aitouny, 2023) highlighted the relevance of recognition, cultural context, and belonging in the experiences of gifted adults with a migration background.

The current study responds to this gap by examining the lived experiences of gifted adults with a migration background. Specifically, it addresses the following research question: How do gifted adults with a migration background experience the interplay between recognition, development, and identity across the lifespan?

## 2. Materials and Methods

### 2.1. Research Design

This study employed a qualitative research design to explore how giftedness is experienced by adults with a migration background. Given the exploratory nature of the research question and the focus on lived experience, a qualitative approach was chosen to capture the complexity of participants’ meaning-making processes across the lifespan. Rather than treating giftedness as a predefined construct, the study sought to understand how participants themselves articulate their experiences of giftedness. An inductive thematic analysis was used to identify and develop patterns through engagement with the data while remaining grounded in participants’ narratives (Naeem et al., 2023). Although themes were developed inductively, the analysis was informed by relevant theoretical perspectives, enabling a conceptually coherent interpretation without imposing a rigid predefined framework or reducing individual variation.

### 2.2. Participants

Participants were adults living in the Netherlands between the ages of 18 and 65 who recognized in themselves in characteristics commonly associated with giftedness and had a migration background.

Participants were recruited through purposive sampling using social media platforms, professional networks, and community-based channels targeting individuals with experiences related to giftedness and migration. Eligibility criteria included having a migration background, proficiency in the Dutch language, and self-recognition in characteristics commonly associated with giftedness. These characteristics included high cognitive ability, strong intellectual curiosity, heightened intensity of experience, advanced cognitive development at an early age, or an independent and complex way of thinking. Some participants reported early educational indicators associated with giftedness, such as grade skipping or participation in enrichment programs.

Self-recognition was used as an inclusion criterion because the study focused on participants’ lived experiences and meaning-making rather than on independently establishing giftedness through formal assessment. Research on gifted adults has highlighted the importance of attending to lived experience and the conceptual diversity in how adult giftedness is defined and studied (Brown et al., 2020). Brown et al. (2020) further emphasize the importance of clearly articulating how giftedness is conceptualized and how participants are selected. In the present study, self-recognition was used as an inclusion criterion relevant to the research question and was not considered equivalent to independently verified giftedness. Similarly, Deligeorges et al. (2022), in a study of self-identified gifted adults, used self-identification because their research focused on inner experience and building self-knowledge, while explicitly acknowledging the potential for sampling bias associated with this approach. Self-recognition in the present study was therefore treated as a sampling criterion relevant to the research question and not as equivalent to independently verified giftedness.

The final sample consisted of 30 participants, including 4 men and 26 women, aged 23 to 65 years, with a mean age of 44 years. Participants were recruited from several regions in the Netherlands, including Zuid-Holland, Gelderland, Utrecht, and Noord-Brabant. The sample included first-, second-, and third-generation individuals with diverse migration backgrounds, including Indonesian/Indisch, Moluccan, Turkish, Moroccan, Surinamese (including Hindustani-Surinamese), Antillean, and former Yugoslavian backgrounds. The sample therefore represented a range of migration backgrounds and generational positions.

The sample encompassed individuals with varied migration histories and educational trajectories, providing an opportunity to explore how giftedness was experienced and recognized across different sociocultural contexts. None of the participants reported having been formally identified as gifted during childhood. Some participants described early indications of advanced learning or recalled that teachers or other adults had noticed that they learned easily or differed from their peers. For some participants, recognition or self-recognition of giftedness occurred later in adulthood. These differences provided insight into how recognition processes were experienced and interpreted across different phases of life.

During the process of data collection and analysis, no substantially new themes relevant to the research question were identified. Although additional nuances were identified during the final stage of data collection, no new main themes were developed. The data were therefore considered to have reached thematic saturation at the level of the main themes, understood as the point at which additional data no longer generated new relevant themes (Saunders et al., 2018).

### 2.3. Data Collection

Data were collected between September and December 2025 through a combination of in-depth, semi-structured interviews and focus groups. In total, 10 individual interviews and four focus groups were conducted. The focus groups consisted of four to six participants, and two participants took part in both a focus group and an individual interview. All sessions lasted approximately 60 to 75 min and were conducted online via Microsoft Teams.

Both the interviews and focus groups were structured around the same central themes, including participants’ educational and developmental experiences, recognition and lack of recognition, belonging, identity and cultural background, and perceived opportunities and barriers. The semi-structured format allowed the order and wording of questions, as well as the depth with which particular topics were explored, to vary according to participants’ narratives. Questions were evaluated throughout the data collection process and, where necessary, rephrased to improve clarity or adapted to allow further exploration of participants’ experiences.

All interviews were conducted by the first author (F.A.). One student researcher was present during one interview, and both student researchers were present during another interview. In these instances, the students could ask follow-up questions related to their respective thesis topics. The remaining interviews were conducted by F.A. alone. All focus groups were moderated by F.A. The student researchers supported the focus groups by taking notes and could ask follow-up questions when participants raised topics relevant to their respective thesis topics. Both student researchers attended the first focus group; in the subsequent focus groups, they attended alternately.

Combining interviews and focus groups allowed personal experiences to be explored alongside shared reflections and interactions between participants. The interviews provided space for more detailed exploration of individual experiences, whereas the focus groups enabled participants to respond to one another and to identify similarities and differences in their experiences. These interactions were also considered analytically, including instances in which participants confirmed, elaborated on, or contrasted one another’s experiences.

With participants’ consent, all sessions were recorded via Microsoft Teams and transcribed. The transcripts were checked against the recordings for accuracy and subsequently anonymized before analysis.

### 2.4. Data Analysis

Data were analysed using an iterative thematic analysis, supported by ATLAS.ti. The analysis began with detailed coding of the interview and focus group transcripts to identify meaningful units of data. Much of the initial coding and preliminary analysis was conducted by the student researchers within the context of their respective thesis topics. The first author subsequently reviewed the analyses, codebooks, and coded transcripts and discussed the preliminary analyses with the student researchers and L.H.

For the present study, the first author further analysed the coded data in relation to the broader research question of this article. Existing codes and categories were reviewed and compared across participants and contexts and, where conceptually related, brought together into broader analytical patterns. This involved moving back and forth between the coded data, individual accounts, and patterns across cases. Particular attention was paid to relationships between participants’ experiences of recognition, belonging, and identity.

Based on this analysis, the first author developed the final themes and subthemes. These were subsequently discussed with L.H. and further refined in relation to the research question and participants’ accounts. Reflexivity was incorporated throughout the analytical process by explicitly considering how the researchers’ different backgrounds, perspectives, and positions in relation to the research topic could influence the interpretation of participants’ accounts. These potential influences were discussed when reviewing and refining the interpretations.

The final analysis resulted in four overarching themes: conditional recognition of giftedness, negotiating belonging and misalignment, identity development across the lifespan, and intersecting identity dimensions.

### 2.5. Ethical Considerations

Ethical approval for the study was obtained from the Ethics Committee Social Science of Radboud University under the Light Track procedure for minimal-risk research (reference number: ECSW-LT-2025-5-2-63329; 2 May 2025). All participants provided informed consent prior to participation, and anonymity and confidentiality were maintained throughout the study. Given the personal nature of some of the topics discussed, including identity, recognition, and experiences of exclusion, interviews and focus groups were conducted with sensitivity and attention to participants’ well-being.

## 3. Results

The analysis showed that participants experienced and narrated giftedness as a context-dependent and relational rather than solely as a fixed individual characteristic. Their accounts highlighted how experiences of giftedness were situated within educational, social, and cultural contexts. Across participants’ life trajectories, three interconnected dimensions were identified: recognition, belonging, and identity. These dimensions were reflected in participants’ accounts across different contexts and stages of life. Table 1 provides an overview of the main themes and subthemes derived from the analysis.

### 3.1. Conditional Recognition of Giftedness

Participants consistently described recognition of their giftedness as contingent on context rather than automatic. In educational settings, for example, they described how linguistic differences, cultural expectations, and behavioural norms were related to whether their abilities were acknowledged or overlooked. Many recalled that their high abilities were not identified during childhood and described experiences in which their abilities or behaviour were interpreted through prevailing expectations about what giftedness should look like. One participant observed, “It’s more about the stereotype of giftedness that we are talking about. The white child, blond hair, blue eyes, glasses… skipping three grades. That’s the image people have” (FG2-P4).

Several participants also described experiences of self-doubt when their abilities remained unrecognized. At the same time, participants did not always attribute these experiences specifically to their migration background or giftedness. As one participant reflected, “I don’t know whether it was because of my migration background or giftedness. I can’t really distinguish between the two” (FG4-P3).

A recurring element in the narratives was the perceived need to prove one’s abilities to be taken seriously. This was described not only in educational settings but also later in professional life. One participant, reflecting on entering a senior professional environment where she initially felt ignored, explained, “At some point I really had to prove myself with facts” (FG4-P4). Recognition was therefore described as requiring additional effort or validation rather than occurring automatically. Across these accounts, whether participants felt recognized as capable depended, at least partly, on how their abilities were perceived and acknowledged within particular contexts.

### 3.2. Negotiating Belonging and Misalignment

Experiences of belonging and lack of fit were identified as a central theme in participants’ accounts. Many described a longstanding sense of being different across cultural, social, and intellectual contexts. They described navigating different environments, including home, school, peer groups, and broader society, in which expectations could differ. What was valued or accepted in one context was not necessarily understood or appreciated in another. Participants described adjusting their behaviour and self-presentation in response to these different expectations. One participant captured this experience by explaining, “I can feel at home everywhere and at the same time nowhere completely. I am a chameleon. I can blend into different environments and switch between them” (I-P4).

For some participants, this ability to adapt was described both as a capacity and as a source of tension. Moving between contexts could involve continuously assessing what behaviour or way of communicating was appropriate. Participants described this particularly in relation to differences between expectations at home and those encountered in Dutch social or educational contexts. In these accounts, belonging was therefore not experienced as fixed or self-evident, but as something negotiated across different environments. For some, adapting to these environments was also accompanied by experiences of invisibility or of not being able to fully express different aspects of themselves.

### 3.3. Identity Development Across the Lifespan

Participants described their understanding of themselves as changing over time, particularly as they came to recognize giftedness as a possible explanation for experiences that had previously been difficult to understand. Giftedness had generally not been part of their self-concept during childhood or adolescence, as none of the participants had been formally identified as gifted during childhood. For many, recognition of giftedness occurred later in adulthood and was described as prompting them to reconsider earlier experiences.

This retrospective reinterpretation could involve both relief and grief. One participant described how a therapist’s suggestion that she might be gifted challenged her longstanding perception of herself as less capable: “I cried for two years because things fell into place.… Now I understand everything.… It is new, fresh, and explains so much, but it also brings a lot of grief” (FG1-P4).

Participants described revisiting earlier experiences of feeling different, being misunderstood, or adapting to others in light of this later recognition. For some, periods of burnout or other difficulties formed part of these retrospective accounts, although the trajectories and circumstances differed between participants.

Participants also described identity development as an ongoing process in which different aspects of themselves could be reconsidered and brought together. Rather than presenting identity as fixed, some described becoming more aware of how giftedness, cultural background, family experiences, and other aspects of their lives related to one another. FG1-P4 used the metaphor of a lasagne to describe this process: “I see myself as a lasagne. We consist of layers. Over the past four years, I have been actively identifying and integrating each layer” (FG1-P4). Across these accounts, later recognition of giftedness provided participants with an additional perspective from which to interpret earlier experiences and to continue constructing their sense of self.

### 3.4. Intersecting Identity Dimensions

In addition to cultural background and giftedness, some participants described other dimensions of identity that contributed to how they understood their experiences. Adoption was one such dimension. One participant explained that she had long attributed her sense of being different primarily to being adopted but later came to understand giftedness as an additional layer: “I always felt different, and I always attributed that to my adoption.… But since coming into contact with gifted people, I have come to see that there is another layer on top of that” (I2-P2).

Religion and spirituality were also meaningful for some participants. Rather than being described solely as another source of difference, faith could provide a framework through which participants understood themselves and their experiences. One participant described this simply: “Prayer gives me peace. Faith gives me strength” (I-P1).

These experiences were not shared by all participants, but they illustrate how giftedness and cultural background could be understood alongside other dimensions of identity. Participants’ accounts of adoption, religion, and spirituality therefore point to the layered nature of identity and to the different ways in which they made sense of belonging and difference.

### 3.5. Summary of Findings

In sum, participants’ accounts highlighted the interconnected roles of recognition, belonging, and identity across different stages and contexts of their lives. Recognition of giftedness was described as context-dependent, while belonging was often negotiated across educational, social, cultural, and professional environments. Later recognition of giftedness provided participants with an additional perspective from which to reinterpret earlier experiences and understand their sense of difference. At the same time, experiences related to adoption, religion, and spirituality illustrate that giftedness and migration background formed part of a broader and layered process of identity development.

## 4. Discussion and Conclusions

This study builds on a growing shift in giftedness research from viewing high ability primarily as an individual characteristic toward understanding gifted development in relation to the contexts in which abilities are recognized, supported, and given meaning. Recent scholarship on giftedness, migration, and displacement has similarly emphasized more systemic and context-sensitive approaches to talent development (Al-Hroub & Ziegler, 2026; Bicakci & Ziegler, 2026). The present study extends this emerging body of work beyond childhood identification and educational provision by examining the retrospective accounts of adults whose experiences of giftedness have unfolded across different sociocultural contexts and stages of life. Their accounts highlight the interconnectedness of recognition, belonging, and identity across the lifespan.

A central contribution of this study concerns the conditional nature of recognition. Participants’ accounts suggest that recognition of giftedness depended not only on individual ability, but also on the institutional and sociocultural contexts in which that ability was interpreted. This aligns with research documenting inequities in the identification of high-ability students from minoritized and migration backgrounds (Ford, 2014; Ford et al., 2026; Gray & Gentry, 2024; Parekh et al., 2018; Ricciardi et al., 2020). Recent Dutch research similarly shows discrepancies between measured high ability and teacher and parent nominations, with identification varying according to factors including language and migration background (Hovinga & Cramer, 2026). The present study extends this literature by showing how adults retrospectively connect experiences of recognition and non-recognition across educational and professional contexts with their developing understanding of themselves and their giftedness. Recognition is therefore conceptualized in these accounts not merely as an identification event but as a context-sensitive process whose meaning can be revisited across the lifespan.

In addition, the findings bring belonging more centrally into the understanding of gifted development. Participants described experiences of misalignment across cultural, social, and intellectual contexts, illustrating the complexity of navigating environments with differing expectations. Belonging was characterized in their accounts not as a static state, but as something negotiated across contexts and closely connected to experiences of recognition and contextual fit. This resonates with research on migration and identity in which belonging is conceptualized as dynamic and context-dependent (Geurts et al., 2021; Schachner et al., 2018). Recent research among gifted adults in a context of forced displacement similarly highlights the relevance of belonging and identity reconstruction in understanding their experiences (Ogurlu et al., 2026). The present findings extend this perspective to gifted adults with a migration background by highlighting how experiences of recognition, misalignment, and belonging can be understood in relation to one another. Belonging thus provides a relational lens for considering how individuals experience their place within the different contexts in which their abilities and identities are expressed.

The findings further underscore the value of a lifespan perspective. For many participants, recognition of giftedness occurred later in adulthood and provided a new interpretive frame through which earlier experiences could be reconsidered. Participants described revisiting experiences of feeling different, being misunderstood, or adapting to others in light of this later recognition. This is consistent with research emphasizing the continued relevance of giftedness and identity development in adulthood (Rinn, 2024; Rinn & Bishop, 2015). Importantly, the present findings do not establish that lack of recognition earlier in life caused particular outcomes in adulthood. Rather, they show how later recognition can become part of the way individuals reinterpret earlier experiences and develop their understanding of themselves over time. A lifespan perspective therefore makes visible not only when giftedness is recognized but also how the meaning attributed to giftedness and its recognition may change across different stages of life.

Importantly, participants’ accounts also need to be understood in relation to broader structural conditions. Research on gifted development has shown that opportunities for recognition and support are not equally available across sociocultural contexts (Ford, 2014; Parekh et al., 2018; Preckel et al., 2020; Subotnik et al., 2012). Recent systemic approaches similarly emphasize that talent development depends not only on individual resources, but also on the availability and accessibility of educational, social, and institutional resources (Bicakci & Ziegler, 2026). The present findings complement this perspective by drawing attention to how such contexts are experienced by adults: not only as environments that provide or restrict opportunities, but also as settings in which abilities are interpreted and recognition and belonging are negotiated. This suggests that sociocultural and institutional contexts should be considered as integral to understanding gifted development rather than as background conditions surrounding an individual developmental process.

The presence of additional identity dimensions, including adoption, religion, and spirituality, further illustrates the layered nature of identity development. Participants’ accounts show how these dimensions could become part of the ways in which they understood their experiences of giftedness, belonging, and difference. Rather than suggesting that giftedness and migration background operate as uniform or isolated categories, these findings point to the importance of considering multiple dimensions of identity and the different meanings they may acquire across contexts and over time. This is consistent with perspectives that situate giftedness within broader processes of identity development and social positioning (Parekh et al., 2018; Rinn, 2024).

From a theoretical perspective, the findings are consistent with ecological models of development (Bronfenbrenner, 1979; Bronfenbrenner & Morris, 2006) and talent development frameworks that emphasize interactions between individuals and their environments (Preckel et al., 2020; Subotnik et al., 2012). They also complement more recent systemic approaches that explicitly foreground the role of contextual conditions in gifted development, including in migration contexts (Bicakci & Ziegler, 2026). The present study adds to these perspectives by highlighting that sociocultural contexts are not only environments in which opportunities and resources are available or constrained, but also contexts in which abilities are interpreted, recognition is negotiated, and experiences of belonging and identity acquire meaning. This suggests that theoretical accounts of gifted development may benefit from greater attention to recognition, belonging, and identity as relational and context-sensitive processes across the lifespan.

Taken together, the findings support a relational and lifespan-oriented conceptualization in which recognition, belonging, and identity development are understood as interconnected processes. Participants’ accounts illustrate how these processes can acquire different meanings across sociocultural contexts and stages of life, without suggesting a fixed sequence or causal relationship between them. Considering these dimensions in relation to one another makes visible connections that may be less apparent when recognition, belonging, and identity are examined separately. This perspective therefore complements existing developmental and systemic approaches by drawing attention to how giftedness is socially recognized and personally understood across the lifespan.

Several limitations should be considered. First, the retrospective nature of the data introduces the possibility of recall bias (Bell & Bell, 2018), as participants reflected on experiences that in some cases occurred many years earlier. Second, the qualitative design and purposive sample limit the transferability of the findings beyond the participants and contexts studied. The sample was also predominantly female and included participants with diverse migration backgrounds, limiting conclusions about experiences associated with particular gender or migration-background groups. In addition, participants recognized in themselves in characteristics commonly associated with giftedness. This self-selection means that individuals who do not recognize or describe themselves in these terms are likely underrepresented. At the same time, the use of self-recognition as an inclusion criterion made it possible to include adults whose experiences of giftedness were not necessarily tied to formal identification, which was consistent with the study’s focus on lived experience.

Future research could build on these findings by adopting longitudinal designs to examine how recognition, belonging, and identity develop and relate to one another over time. Such studies could prospectively examine whether and how the timing of recognition is associated with later developmental experiences and outcomes. Comparative research across different sociocultural contexts could further clarify which patterns are context-specific and which are observed across different groups and settings. In addition, intervention research could examine how culturally responsive approaches to identification and support affect experiences of recognition and belonging among individuals from diverse backgrounds. Together, these directions could contribute to a more context-sensitive and lifespan-oriented understanding of gifted development.

In conclusion, this study contributes to a more context-sensitive and lifespan-oriented understanding of gifted development by bringing the experiences of adults with a migration background into a literature that has largely focused on childhood and educational settings. The findings highlight recognition, belonging, and identity as interconnected processes whose meanings may change across contexts and over time. Understanding gifted development therefore benefits from attention not only to individual ability and opportunities for its development, but also to how giftedness is recognized and understood within the sociocultural contexts in which individuals live.

## Figures and Tables

**Table 1:** Overview of themes and subthemes identified in the data.

Theme	Subthemes	Analytical Description
Conditional recognition of giftedness	Context-dependent recognition	Recognition of giftedness is situational and uneven, shaped by institutional and sociocultural contexts rather than stable individual traits
	Need for validation	Participants describe a recurring need to demonstrate ability to be acknowledged
	Normative framing of ability	Dominant norms influence which expressions of giftedness are recognised or overlooked
Negotiating belonging and misalignment	Movement across contexts	Participants navigate multiple cultural and social environments with differing expectations
	Experienced lack of fit	Persistent sense of not fully belonging within educational and professional settings
	Asynchronous alignment	Mismatch between cognitive, social, and contextual demands complicated both participation and recognition
Identity development across the lifespan	Delayed identification	Giftedness is often recognised retrospectively, reshaping earlier self-perceptions
	Reframing lived experiences	Past experiences are reinterpreted through the lens of giftedness
	Ongoing identity construction	Identity development was understood as a continuous, context-sensitive process across participants’ accounts.
Intersecting identity dimensions	Adoption and origin	Adoption introduces additional layers of reflection on belonging and self-understanding
	Religion and meaning- making	Religious frameworks contribute to meaning-making and identity positioning
	Layered identity dynamics	Multiple identity dimensions interact, intensifying developmental complexity

## Data Availability

The data are not publicly available due to privacy and confidentiality considerations. Access to the data may be considered in accordance with participants’ informed consent and the applicable institutional and ethical requirements.
